# Chemistry of the Fe_2_O_3_/BiFeO_3_ Interface in BiFeO_3_ Thin Film Heterostructures

**DOI:** 10.3390/ma3125274

**Published:** 2010-12-14

**Authors:** Miryam Arredondo, Quentin M. Ramasse, Kashinath Bogle, Valanoor Nagarajan

**Affiliations:** 1 School of Materials Science and Engineering, University of New South Wales, Sydney NSW 2052, Australia; E-Mails: arredond@mpi-halle.mpg.de (M.A.); kashinath.bogle@unsw.edu.au (K.B.); 2 SuperSTEM Laboratory, STFC Daresbury, Keckwick Lane, Daresbury WA4 4AD, UK; E-Mail: qmramasse@superstem.org

**Keywords:** mutliferroic BiFeO_3_, perovskite, Z-contrast imaging, EELS

## Abstract

We investigate the interfacial chemistry of secondary Fe_2_O_3_ phases formed in a BiFeO_3_ (BFO) layer in BFO/ La_0.67_Sr_0.33_MnO_3_ (LSMO)/SrTiO_3_ (STO) heterostructures. A combination of high-resolution spherical aberration corrected scanning TEM and spectroscopy results, reveals that specific chemical and crystallographic similarities between Fe_2_O_3_ and BFO, enable the BFO layer to form a facile host for Fe_2_O_3_.

## 1. Introduction

Perovskite Bismuth Iron Oxide (BiFeO3, BFO) has attracted immense interest in recent times as, not only, a multiferroic [1,2] but also a Pb-free piezoelectric candidate, with attractive electromechanical properties [3,4]. However, Bismuth (Bi) is quite similar to Lead (Pb) in terms of its chemical stability—it is volatile under high temperatures and vacuum. Consequently, BFO thin film deposition requires the use of Bi-excess targets or precursors to compensate for the Bi loss at nominally employed high growth temperatures [5]. Moreover BFO has a very narrow thin film deposition window to obtain phase pure BFO thin films, which necessitates a rigid control in terms of the precursor chemistry, temperature and oxygen pressure, to synthesize the films. For example, incorrect Bi concentration in the targets can create secondary phases, such as Fe_2_O_3_ [6,7], while improper oxygen environments during laser ablation can lead to pockets of Bi_2_O_3_ [6]; on the other hand, high growth temperatures can also lead to cation diffusion across the thin film interfaces [8]. Additionally, it has been found that oxygen vacancies could induce significant leakage current [9,10]. Among the secondary phases that can be created during the BFO deposition process, there has been a significant interest in Fe_2_O_3_ phases found in BFO thin films. A highly controversial issue is whether or not parasitic Fe_2_O_3_ is the primary reason for enhancement of magnetic properties in BFO [2]. The presence of the Fe_2_O_3_ phase also complicates the study of BFO thin films [6,11] as a model multiferoic material. On the other hand, studies have shown that Fe_2_O_3_ embedded in BFO can be considered to be a new type of multiferroic nanocomposite material [7]. For example, it has been reported that the presence of α- and γ-Fe_2_O_3_ phases enhances the BFO strain relaxation and magnetization [9]. These previous reports on the associated impact of Fe_2_O_3_ on the properties of BFO, claim that detailed high‑resolution studies are needed in order to understand the nature of the interface between the Fe_2_O_3_ phase and the BFO film. The objective of this study is to understand the chemical and structural features of an interface between BFO and Fe_2_O_3_ and, hence, reveal why this system is so facile for such nanocomposite synthesis.

## 2. Results and Discussion

**Figure 1:**
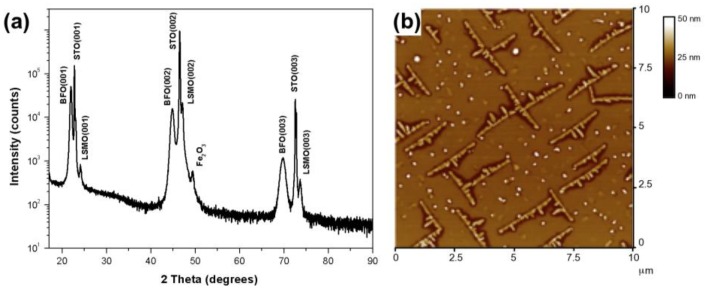
(**a**) XRD data. The XRD pattern shows an extra peak around ~49 degrees that is identified as Fe_2_O_3_. (**b**) Atomic force microscope scans of this sample, reveals the Fe_2_O_3_ phase forming a criss-cross network of elongated islands nucleated within the BFO matrix.

For this study a 50 nm thick BFO layer was deposited on a 50 nm thick La_0.67_Sr_0.33_MnO_3_ (LSMO) layer, used as a bottom electrode on a SrTiO_3_ (STO) substrate via pulsed laser deposition (PLD). Conditions were deliberately altered (see experimental details) to induce the formation of secondary Fe_2_O_3_ phases. The BFO/LSMO/STO heterostructure was chosen as it couples a multiferroic (BFO) with a ferromagnet electrode (LSMO) on an oxide substrate (STO); this is an oxide system that has garnered significant recent attention [1,12,13,14,15]. Figure 1(a) displays the X-ray diffraction (XRD) graph for this sample. It confirms that the desired perovskite BFO, LSMO and STO phases are present in the samples. However, the XRD also shows an extra peak on the right shoulder of the LSMO (002) peak, at ~49 degrees, which is identified to be a secondary phase: α-Fe_2_O_3_ (024). Atomic force microscope scans of this sample (Figure 1(b)) reveals that the Fe_2_O_3_ phase forms a criss-cross network of elongated islands nucleated within the BFO matrix.

**Figure 2:**
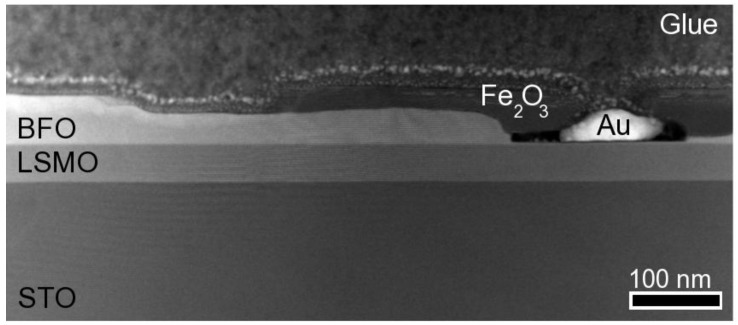
[001] Z-contrast overview image.

Z-contrast imaging for the heterostructure investigated here (Figure 2) indicates that the BFO layer is not uniform but rather has variable thickness; it being 50 nm in the thickest areas and just a few nanometers in the thinnest areas. In regions where the BFO is ultra-thin (or barely present) the region has voids that are occupied by Fe_2_O_3_. The void shown in the figure also contains gold that has leaked inside from the protective coating that was used for sample preparation for transmission electron microscopy. The voids have a layer of Fe_2_O_3_ surrounding the gold above the BFO layer, which indicates that the Fe_2_O_3_ phase is not uniform. This strongly hints that in those areas, the ablated BFO has broken down into Fe_2_O_3_ and Bi_2_O_3_ (that is likely to have volatized).

**Figure 3:**
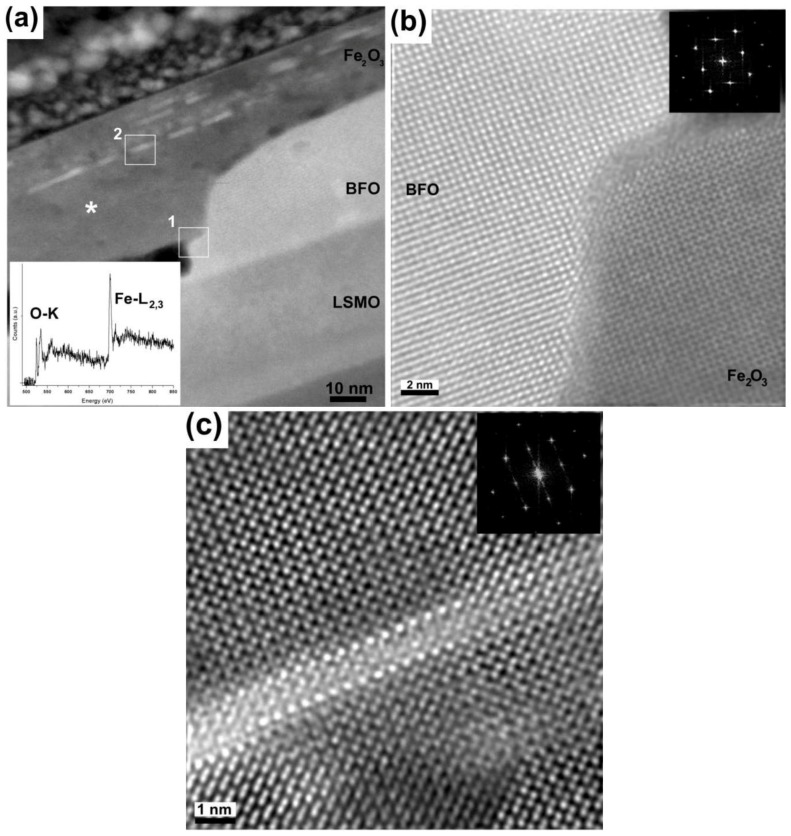
[001]_BFO_ Z-contrast images. **(a)** Overview showing the pocket area and the Fe_2_O_3_ layer containing bright inclusions. Its inset contains an EEL spectrum from the area marked by the asterisk, confirming the presence of Fe; **(b)** Area marked as 1 in (a), showing the BFO and Fe_2_O_3_ merger with their respective diffraction patterns as insets; **(c)** Area marked as 2 in (a) with its corresponding diffraction pattern, showing an inclusion.

Electron energy loss spectroscopy (EELS) was performed to confirm that the chemical nature of the layer on top of the BFO, and ascertain that it is indeed the Fe_2_O_3_ found in the XRD pattern (Figure 1(a)). An EEL spectrum (inset in Figure 3(a)) was acquired from the area shown in Figure 3(a) (marked by an asterisk). It shows the two sharp peaks, or ‘white lines’, around 708 eV, which are characteristic of the Fe-L_2_,_3_ edge as well as a very distinctive O-K edge (onset at about 530 eV), comprising three main peaks. The overall shape and fine structure of these edges match well with those of reference Fe_2_O_3_ spectra, as previously reported by Colliex and Kurata for instance [16,17,18]. Furthermore the Z-contrast image in Figure 3(a) reveals two distinct areas of interest: The direct interface between the BFO and Fe_2_O_3_ layers (a subset of which is marked as 1 on the figure) and some bright inclusions (marked as 2), located deep within the Fe_2_O_3_ layer that exhibit an intensity level more similar to that of the BFO phase than of the immediately surrounding Fe_2_O_3_. Figure 3(b) is a high‑resolution [001]_BFO_ Z-contrast image of Area 1 in Figure 3(a), which corresponds to the interface between the Fe_2_O_3_ and BFO phases. The Fourier transform (shown as an inset) reveals that the two phases appear coherent, indicating epitaxial growth. The diffraction spots resemble those of the BFO [001]. For this particular area, it is suggested that Fe_2_O_3_ adopts the BFO structure due to its proximity to the BFO layer: Both the perovksite-BFO and α-Fe_2_O_3_ have a distorted rhombohedral structure and epitaxial growth is therefore strongly favored. Figure 3(c) on the other hand, is an image of Area 2 containing one of the distinctive inclusions highlighted earlier. While the matrix surrounding the bright inclusion is structurally coherent, it is clearly seen from the image itself that the atomic columns forming the edge of the inclusion are not only brighter but also bigger in size than the surrounding Fe columns, closely matching the intensity and diameter of images of Bi columns acquired at the same magnification and in the same conditions within the pure BFO region. Furthermore, upon closer inspection, the walls of the inclusion itself do not follow quite the same structure as the surrounding Fe_2_O_3_, but appear to adopt a tetragonal BFO structure. These observations strongly suggest that the extra phase enclosed within the Fe_2_O_3_ phase is nanoscale BFO inclusions. The Fast Fourier transform of this image (shown as an inset) reveals that even deep inside the Fe_2_O_3_ phase, the structure remains coherent to the bottom BFO layer and the inclusions.

**Figure 4:**
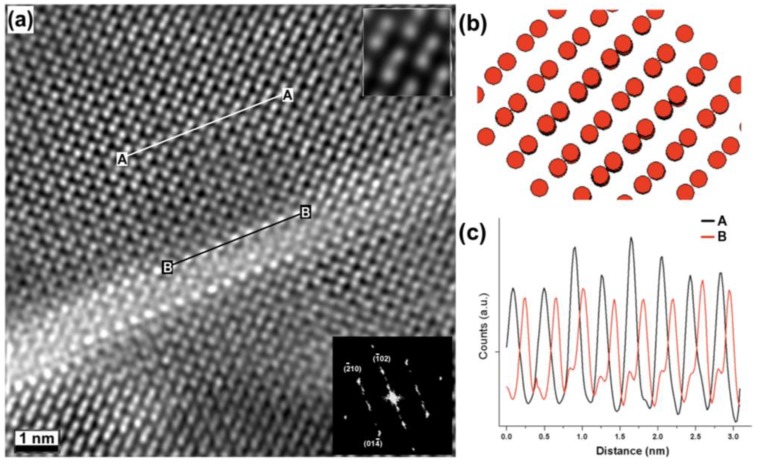
**(a)** Z-contrast image of the Fe_2_O_3_ phase containing a BFO stripe, the upper inset is a filtered image showing the Fe^3+^ doubling of the structure. **(b)** Model of the tilted [241] Fe_2_O_3_ structure, showing the doubling on the Fe^3+^ atomic columns. **(c)** Intensity profiles from (a), measured along the lines marked as A and B.

Further high-resolution imaging was performed to investigate how the inclusions are accommodated inside the Fe_2_O_3_ layer. Figure 4(a) shows how the region surrounding the inclusion has a perovskite-like short order structure (a few unit cells) followed by what was determined to be a near‑[241] α-Fe_2_O_3_ rhombohedral structure (space group $R\bar{3}c$), where the Fe^3+^ cations are projected with a doubling structure. A similar case, where the Fe_2_O_3_ phase was also deviated from the [001]_BFO_, has been previously reported by Murakami *et. al*., [6]. To confirm this structure, a model of the Fe_2_O_3_ structure viewed along the tilted [241] was created using Crystal Maker software (where the O^2-^ atoms are not represented for clarity purposes), shown in Figure 4(b), where the Fe^3+^ doubling is observed. It is clear how this model matches the experimental image. Intensity profiles were measured parallel to the inclusion line, along the c-axis, in order to calculate the distance between Fe doublets and the bright atoms (lines marked as A and B in Figure 4(a)), shown in Figure 4(c). The measurements confirmed a distance of ~0.391 nm for both cases, which is close to that of BFO (0.396 nm [19,20]). Hence, the mismatch between these two phases is very small (~1%). This now explains how the Fe_2_O_3_ structure can easily be accommodated on the BFO structure and its predisposition to adopt the lattice arrangement of the ultrathin BFO inclusions, namely the distorted tetragonal structure.

**Figure 5:**
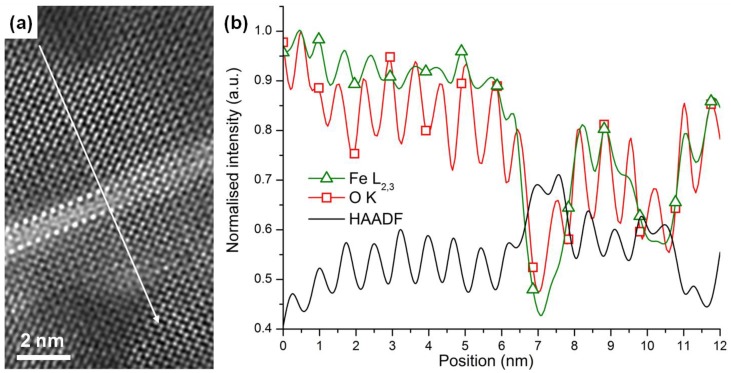
EELS line scan inside the Fe_2_O_3_, across the stripe. **(a)** Z-contrast image, indicating the points from where the EEL spectra were acquired and **(b)** integrated Fe and O intensity as a function of probe position, along with the simultaneously acquired HAADF signal (green line with triangle markers, red line with square markers and solid black line respectively). The Fe-L_2,3_ edge (res. O-K edge) was integrated over a 60 eV (resp. 50 eV) window above its edge onset. In both cases, the background was removed using a power law model.

Point-by-point electron energy loss spectroscopy (EELS) was also performed across the needle‑shaped inclusion inside the Fe_2_O_3_ phase, to gain some insight into its local chemistry. Figure 5(a) is a Z-contrast image of one of the bright inclusions: 50 EEL spectra were acquired serially from top to bottom along a ~12nm line crossing the structure, as indicated on the figure by the white arrow. Figure 5(b) shows the integrated Fe and O EELS intensity as a function of probe position, along with the simultaneously acquired high angular annular dark field (HAADF) signal (green line with triangle markers, red line with square markers, and black solid lines, respectively). The Fe-L_2,3_ edge (res. O-K edge) was integrated over a 60 eV (resp. 50 eV) window above its edge onset. In both cases, the background was removed using a power law model. A striking feature of this intensity profile is the marked intensity decrease in the O-K and Fe-L_2,3_ edges coinciding precisely with the bright needle-shape inclusion, as evidenced by the simultaneous increase in HAADF intensity. This result points to a lower Fe content and/or the presence of oxygen vacancies [18] within the inclusion. Although no EELS spectrum was acquired to confirm categorically the presence of Bi atoms (the Bi-M edge energy being too high for practical acquisition on the instrument used for this study), the drop in Fe intensity, along with the bright contrast of the atomic columns, strongly suggests that the inclusions are Bi-rich. Combined with our prior structural analysis based on the HAADF images, it is therefore likely that those needle-shaped inclusions mark the thwarted onset of BFO growth, possibly due to a slight change in the pressure/temperature conditions in the PLD chamber.

## 3. Experimental Section

BiFeO_3_ (BFO)/La_0.67_Sr_0.33_MnO_3_ (LSMO)/SrTiO_3_ (STO) heterostructures were deposited via PLD. The films were deposited at 900 °C, 100 mTorr O_2_ with a laser fluence of 2 J.cm^−2^ for LSMO and 900 °C, 10 m Torr O_2_ with a laser fluence of 1.6 J.cm^−2^ and a frequency of 10 Hz, then cooled at 20° min^−1^ under a 200 Torr O_2_ atmosphere.

Cross-sectional TEM samples were prepared by standard mechanical tripod polishing and ion beam thinning procedures [21]. The samples were gold-coated prior to TEM sample preparation in order to protect the top layer. Except where otherwise specified, Z-contrast imaging and electron energy loss spectroscopy (EELS) were carried out using an aberration-corrected, dedicated STEM VG HB UX operated at 100kV and equipped with an Enfina parallel EELS detector. For all data presented here, the beam convergence semi-angle was 21 mrad with HAADF detector inner and outer radii of 70 and 210 mrad, respectively. Z-contrast images were used to position the ~1 Å probe on specific atomic positions or line scans, with a dispersion of 0.2 to 0.3 eV per channel and a collection semi-angle of 19 mrad. Except where specified, Z-contrast image were processed using a probe deconvolution algorithm based on maximum entropy methods in order to minimize the noise levels [22].

## 4. Conclusions

The interfacial structure and chemistry of secondary Fe_2_O_3_ phase found in BFO/LSMO heterostructure is investigated. HRTEM and STEM images reveal that Fe_2_O_3_ pockets can be easily accommodated by the host BFO phase, due to a similar crystallographic structure and close epitaxial registry. The observation of Fe-deficient bright needle-shaped inclusions within the Fe_2_O_3_ phase also suggests that minute changes in the growth conditions could trigger BFO growth, although in our case this growth was not sustained. This, combined with previously mentioned structural similarity, could explain why Fe_2_O_3_ is so easily formed during BFO thin film synthesis, and possibly open new routes for creating hybrid nanoscale Fe_2_O_3_-BFO heterostrucures.
